# Ethical Leadership and Leaders’ Emotions: An Identity-Based Perspective on Moral Pride

**DOI:** 10.3390/bs16101728

**Published:** 2026-09-23

**Authors:** Qianyu Sha, Qian Guo

**Affiliations:** Business School, Beijing Normal University, Beijing 100875, China; 202421030033@mail.bnu.edu.cn

**Keywords:** ethical leadership behavior, moral pride, core self-evaluation, burnout, positive emotions, negative emotions

## Abstract

Ethical leadership research has predominantly focused on its consequences for followers and organizations. Adopting an actor-centric, identity-based perspective, this research examines how ethical leadership behavior relates to leaders’ own emotional experiences through moral pride and how this process varies with core self-evaluation and burnout. Three studies using complementary methods tested the proposed relationships. Study 1 showed that unethical leadership behavior significantly reduced moral pride relative to the control and ethical conditions, whereas ethical behavior did not significantly increase moral pride relative to the control condition; CSE moderated the effect involving unethical behavior. Study 2 found significant indirect effects of ethical leadership behavior on positive and negative emotions through moral pride, with CSE moderating the relationship between ethical leadership behavior and moral pride. Study 3, using experience sampling, revealed exploratory conditional patterns across burnout levels: under relatively low burnout, the indirect relationship through moral pride was more evident for positive emotions, whereas under relatively high burnout, the negative indirect relationship with negative emotions was more pronounced. Together, these findings specify moral pride as a self-conscious emotional mechanism and highlight the relevance of individual and resource-related conditions in this process.

## 1. Introduction

Ethical leadership behavior has been a central topic in organizational behavior research for the past two decades. Recurring corporate scandals and ethical failures continue to underscore the importance of leaders’ responsibility for maintaining ethical standards in organizations (Farndale et al., 2025; Amory et al., 2024; Mion et al., 2023; Sajjad et al., 2024; Wang et al., 2024). Compared with lower-level employees, managers and executives are also held to higher moral expectations (Greenbaum et al., 2021; Mitchell et al., 2024). Accordingly, understanding the consequences of ethical leadership is important not only for followers and organizations but also for leaders themselves.

Although emerging scholarship has begun to examine the affective mechanisms through which ethical leadership shapes subordinates’ behavior and organizational outcomes (Aghighi, 2020; Mao et al., 2020), the majority of research has focused on its consequences for followers, teams, and organizations (Eluwole et al., 2022; Schwepker & Dimitriou, 2021; Torlak et al., 2021; X. M. Xu & Yu, 2021). Comparatively less attention has been paid to how ethical leadership behavior influences the emotional experiences of leaders themselves (Yip & Lee, 2022). Building on prior actor-centric research on the consequences of ethical leadership (Z. Xu & Ju, 2024), the present study extends this line of inquiry by shifting attention from performance-related outcomes to leaders’ own positive and negative emotional experiences. This focus is important because leaders’ emotions are consequential not only as affective experiences in their own right but may also have downstream effects on subordinates through processes such as affective contagion, ultimately shaping team functioning and organizational outcomes (Farooq & Khan, 2026; Xerri et al., 2023). Investigating how ethical leadership behavior relates to leaders’ own emotional states is therefore both theoretically meaningful and practically important. Research on moral identity and self-conscious emotions provides a useful theoretical lens for understanding these intrapersonal emotional consequences (e.g., Blasi, 1984; Tracy & Robins, 2007b).

We propose that moral identity provides an important theoretical basis for understanding the intrapersonal effects of ethical leadership. Moral identity refers to the extent to which being a moral person is central to an individual’s self-concept (Aquino & Reed, 2002). Importantly, the centrality of moral identity varies across individuals, and engaging in ethical leadership behavior does not necessarily imply that a leader possesses a highly central moral identity. Moral identity theory suggests that individuals evaluate their conduct in relation to moral standards that are relevant to their self-concept. Accordingly, when leaders perceive their ethical conduct as congruent with their moral self-concept, such perceived congruence can trigger a self-evaluative process. A positive appraisal arising from this process may elicit self-conscious emotions, most notably moral pride—a feeling of satisfaction and self-worth derived from seeing oneself as having acted morally (Tangney et al., 2007b). Moral pride, in turn, may function as an affective and cognitive resource (cf. Conservation of Resources Theory; Hobfoll, 1989), reinforcing a positive self-view and potentially contributing to more positive and fewer negative emotional experiences. Thus, our framework shifts attention from leaders merely reacting to external events or catching emotions from others toward an internal, identity-relevant appraisal process. The strength of this emotional response, however, may vary across individuals. In particular, core self-evaluation (CSE) captures fundamental evaluations of one’s own worth and capabilities (Schilpzand et al., 2025). Leaders with different levels of CSE may evaluate themselves differently in similar ethical contexts, which in turn may contribute to divergent emotional responses (Mao et al., 2020; Son & Pak, 2024).

In addition to stable individual traits, situational resource conditions may further shape leaders’ emotional responses to ethical conduct (Santiago-Torner et al., 2024). Conservation of Resources Theory suggests that individuals’ responses to emotional experiences may depend on their existing resource conditions, such that resource depletion can constrain their capacity to benefit from positive psychological resources (Hobfoll, 1989; Liao et al., 2025). Burnout reflects a state of depleted psychological resources and may therefore condition the extent to which leaders can translate moral pride into subsequent emotional experiences (Khan et al., 2024; Santiago-Torner et al., 2024). Specifically, although moral pride may constitute a positive affective resource, leaders experiencing higher burnout may have fewer available resources to capitalize on this positive self-conscious emotion, potentially weakening its association with subsequent positive and negative emotional experiences. By examining CSE as a stable individual characteristic that shapes self-appraisal and burnout as a situational resource condition that may be relevant to the emotional consequences of moral pride, we consider individual and resource-related conditions at two distinct points in the proposed process: the initial generation of moral pride and its subsequent association with leaders’ emotional experiences. This provides a more nuanced understanding of the intrapersonal emotional process associated with ethical leadership behavior.

Building on this foundation, the present research examines how ethical leadership behavior is related to leaders’ positive and negative emotional experiences. We conducted three complementary studies. Study 1 was an experiment with 205 participants. Study 2 used a two-wave matched field design with 132 leaders, and Study 3 used an experience-sampling design involving 52 leaders who completed repeated assessments over 10 workdays. Across these studies, we address three related research questions: (1) How is ethical leadership behavior related to leaders’ positive and negative emotional experiences? (2) Does moral pride serve as a psychological mechanism linking ethical leadership behavior to these emotional experiences? (3) How do individual and situational conditions shape these relationships?

Addressing these questions contributes to the ethical leadership literature in three important ways. First, building on prior actor-centric research, we extend understanding of the intrapersonal consequences of ethical leadership by shifting attention to leaders’ own emotional experiences. Second, we examine moral pride as a self-conscious emotional mechanism linking ethical leadership behavior to leaders’ broader positive and negative affective experiences, thereby providing a more differentiated account of the emotional consequences of ethical conduct. Third, we examine complementary individual and situational conditions by considering CSE as a stable individual characteristic that may shape the relationship between ethical leadership behavior and moral pride, and burnout as a resource-related state that may be relevant to the relationship between moral pride and subsequent emotional experiences.

### 1.1. Ethical Leadership Behavior and Emotional Experience

Ethical leadership research has traditionally centered on its interpersonal consequences (e.g., Mishra et al., 2022), with comparatively less attention devoted to the intrapersonal emotional experiences of leaders themselves. To explain how leaders may emotionally experience their own ethical conduct, we draw on moral identity theory (Aquino & Reed, 2002; Blasi, 1984) and self-conscious emotion theory (Tracy & Robins, 2007b). Moral identity captures the extent to which being a moral person is important to an individual’s self-concept, and this centrality varies across individuals (Aquino & Reed, 2002). Existing research further suggests that moral identity is differentiated across social roles, such that the moral identity content most relevant to a salient role can become particularly influential in guiding moral responses within that context (Hannah et al., 2020). Accordingly, ethical conduct enacted in a leadership role may render morally relevant aspects of the self more salient and provide a basis for evaluating one’s conduct against personally meaningful moral standards. We contend that the emotional significance of ethical leadership behavior for the actor stems from this self-evaluative process.

Central to this identity-based perspective is the moral self-schema, which refers to the cognitive representation of one’s moral identity. Individuals are motivated to act in ways that affirm aspects of the self that are salient and important to their self-concept. A substantial body of empirical research has shown that stronger moral identity is associated with greater engagement in prosocial and ethical behaviors and with lower levels of antisocial behavior and moral disengagement (Aquino & Reed, 2002; Reynolds & Ceranic, 2007; Detert et al., 2008; Moore et al., 2019; Mesdaghinia et al., 2022). When leaders engage in ethical behavior, such conduct can be appraised in relation to their morally relevant self-standards and, when perceived as congruent with those standards, can constitute a self-verifying experience (Stets & Carter, 2011; Hannah et al., 2020). According to self-conscious emotion theory, self-relevant events trigger evaluations against one’s internal standards, and favorable evaluations of one’s conduct can elicit discrete self-conscious emotions. In the moral domain, perceiving that one has acted in accordance with relevant moral standards can elicit moral pride, a positive self-conscious emotion arising from favorable evaluations of one’s moral conduct (Tangney et al., 2007b). Research further distinguishes between two facets of pride: authentic pride is behavior-focused and can promote future prosocial and ethical actions, whereas hubristic pride is more self-focused and may lead to arrogance and moral self-licensing (Tracy & Robins, 2007a, 2014; Mercadante et al., 2021). Accordingly, pride arising from ethical conduct is more closely aligned with moral pride and the behavior-focused characteristics of authentic pride (Tangney et al., 2007a, 2007b). Moral pride can arise from alignment with internal moral standards and may also be reinforced when individuals perceive their actions as socially valued and anticipate positive evaluations from others (Tracy & Robins, 2014; Hannah et al., 2020).

This experience of moral pride is more than a fleeting positive feeling; it can serve as an affective resource (Fredrickson, 2001; Hobfoll, 1989). Extending self-conscious emotion research, recent studies have begun to capture the within-person, daily fluctuations of these emotions, showing that they can be triggered by dynamic behaviors and events in daily life (Tang et al., 2020). By affirming a positive evaluation of one’s moral conduct, moral pride may contribute to psychological resources, foster a sense of self-integrity, and help build enduring personal resources. Moral pride can be viewed as an internally experienced positive emotional resource, which differs from external resources arising from social interactions, such as follower trust and respect. This emotional resource may contribute to leaders’ subsequent affective experiences. The resources associated with moral pride may help sustain broader positive affect, such as feeling enthusiastic and inspired, and may also buffer against negative affect, such as feeling distressed and upset, over time.

Therefore, we propose that ethical leadership behavior can have intrapersonal emotional consequences for leaders through a self-evaluative process. When ethical conduct is appraised as consistent with morally relevant self-standards, it can elicit moral pride, a discrete self-conscious emotion that may subsequently function as an affective resource contributing to broader positive and negative affective experiences. This reasoning is consistent with behavioral ethics research showing that morally relevant self-processes can shape individuals’ responses to their own conduct (Zhang et al., 2014). Taken together, this perspective provides an actor-centric explanation for how ethical leadership behavior may relate to leaders’ own emotional experiences, complementing the predominantly interpersonal mechanisms emphasized in prior ethical leadership research.

Based on the above reasoning, the present study proposes the following hypotheses:

**Hypothesis** **1a.** *Ethical leadership behavior is positively associated with leaders’ positive emotions*.

**Hypothesis** **1b.** *Ethical leadership behavior is negatively associated with leaders’ negative emotions*.

### 1.2. The Mediating Role of Moral Pride

While research has increasingly considered the emotional consequences of ethical leadership, existing studies have largely examined either interpersonal emotional effects on followers or broader intrapersonal affective experiences of leaders. Building on this literature, greater attention should be given to the psychological process through which leaders’ own ethical conduct is associated with their subsequent affective experiences. We focus on moral pride—a discrete, self-conscious moral emotion arising from favorable evaluations of one’s moral conduct (Tangney et al., 2007b)—as a potential emotional mechanism linking ethical leadership behavior to leaders’ broader positive and negative affective experiences.

This focus moves beyond treating moral pride as a simple outcome or a diffuse positive feeling by emphasizing its distinct self-evaluative and motivational properties. Prior research has characterized moral pride as a prescriptive moral emotion that can encourage subsequent norm-consistent behavior (Tangney et al., 2007a, 2007b; Dasborough et al., 2020). In the context of ethical leadership, moral pride reflects a favorable evaluation of one’s own moral conduct and reinforces a positive self-representation (Z. Xu & Ju, 2024). These properties provide a basis for considering moral pride not only as an emotional consequence of ethical conduct but also as a potential mechanism through which ethical leadership behavior relates to leaders’ subsequent affective experiences.

The self-evaluative logic underlying our theoretical perspective helps explain when and why moral pride may arise: when leaders appraise their ethical conduct as congruent with morally relevant self-standards, this perceived self-congruence can elicit moral pride (Tracy & Robins, 2004, 2007a). This mechanism has two features that distinguish it from alternative explanations. First, it is actor-centric and self-conscious. Unlike emotional contagion, which relies on interpersonal influence, moral pride arises from leaders’ evaluations of their own conduct against personally relevant moral standards. Thus, the mechanism emphasizes an internally generated self-evaluative process rather than emotional responses transmitted through social interaction. Consistent with this reasoning, research has shown that self-conscious emotions can be elicited by daily behaviors and events and exhibit meaningful within-person fluctuations (Tang et al., 2020). Second, moral pride provides a theoretical link between a specific self-conscious moral emotion and broader affective experiences. Beyond its immediate emotional experience, moral pride can function as an affective resource (Fredrickson, 2001; Hobfoll, 1989). Research on authentic pride, as distinguished from hubristic pride, further suggests that such pride is associated with prosocial and goal-congruent actions, enhanced self-esteem, prestige, and transformational leadership behaviors (Cheng et al., 2010; Yeung & Shen, 2019). By reinforcing favorable self-evaluations and a sense of integrity, moral pride may contribute to the accumulation of psychological resources and personal resilience (Hobfoll, 1989). From a broaden-and-build perspective (Fredrickson, 2001), these positive emotional and psychological resources can broaden individuals’ cognitive and behavioral repertoires and support subsequent positive affective experiences. At the same time, accumulated resources may provide a buffer against negative affective experiences. Accordingly, moral pride, as a discrete self-conscious moral emotion, may contribute to greater positive affect (e.g., enthusiasm and inspiration) and lower negative affect (e.g., distress and upset) over time.

In essence, moral pride provides an emotional mechanism linking leaders’ ethical conduct to their broader affective experiences. When ethical conduct is favorably evaluated against morally relevant self-standards, the resulting moral pride may function as an affective resource that supports subsequent positive affect and buffers against negative affect. Thus, moral pride helps explain how ethical leadership behavior may be associated with leaders’ subsequent positive and negative emotional experiences. Based on this reasoning, the present study proposes the following hypothesis:

**Hypothesis** **2.** *Moral pride mediates the relationship between ethical leadership behavior and both positive and negative emotions.*

### 1.3. The Moderating Role of Core Self-Evaluation

The extent to which individuals experience moral pride in relation to ethical leadership behavior may vary depending on personal characteristics, with core self-evaluation (CSE) emerging as a potential moderator (Krauss & Orth, 2022; Peláez-Fernández et al., 2022). Even when engaging in similar ethical conduct, individuals may differ in how they interpret and evaluate their own behavior. We propose that CSE moderates the relationship between ethical leadership behavior and moral pride because it shapes how individuals evaluate self-relevant information arising from their own conduct.

Core self-evaluation, as a higher-order personality construct encompassing self-esteem, generalized self-efficacy, emotional stability, and locus of control (Krauss & Orth, 2022; Peláez-Fernández et al., 2022), has been empirically linked to ethical leadership behaviors (X. M. Xu & Yu, 2021). Research indicates that individuals with elevated CSE levels demonstrate enhanced psychological adjustment, greater life control perceptions, and more stable self-concepts, whereas their low-CSE counterparts frequently exhibit self-concept fragility and diminished capability beliefs (Peláez-Fernández et al., 2022). These differences in the stability and positivity of individuals’ self-evaluations provide a basis for expecting CSE to shape their emotional responses to self-relevant ethical conduct.

Building on these premises, we posit that CSE moderates the relationship between ethical leadership behavior and moral pride. Individuals with high CSE generally possess a relatively stable and positive self-concept. For these individuals, positive self-relevant information derived from engaging in ethical conduct may be relatively consistent with their existing favorable self-evaluations and therefore provide less incremental reinforcement, resulting in a comparatively weaker increase in moral pride. In contrast, individuals with lower CSE tend to hold less positive and less stable self-evaluations (Ding et al., 2022; Sun et al., 2023). Consequently, favorable self-relevant information arising from their own ethical conduct may be more salient and psychologically consequential, strengthening the positive evaluation of their behavior and, in turn, their experience of moral pride (Marbut et al., 2025). Accordingly, the positive relationship between ethical leadership behavior and moral pride should be stronger among leaders with lower CSE.

Based on this reasoning, we propose the following hypothesis:

**Hypothesis** **3.** *Core self-evaluation moderates the positive relationship between ethical leadership behavior and moral pride, such that the relationship is stronger for leaders with lower core self-evaluation.*

### 1.4. Burnout as a Resource-Related Condition

Prior research has increasingly recognized that engaging in ethical conduct may yield psychological benefits for leaders themselves, in addition to generating favorable interpersonal consequences (Chughtai, 2023). Such benefits can arise when morally valued conduct is positively self-evaluated, giving rise to self-conscious emotional experiences such as moral pride (Van Tuin et al., 2021). As discussed above, moral pride may subsequently function as an affective resource that contributes to broader positive affective experiences and helps buffer against negative affect. However, the extent to which leaders can derive these broader emotional benefits from moral pride may depend on their available psychological resources.

Drawing on Conservation of Resources Theory (Hobfoll, 1989), we conceptualize burnout as a state characterized by depleted psychological and emotional resources. Although moral pride may itself constitute a positive affective resource, leaders’ ability to build on this resource may vary depending on the extent of their existing resource depletion. When burnout is relatively low, leaders retain greater psychological capacity to sustain and elaborate on the positive self-evaluative experience associated with moral pride (Wu et al., 2022). Under such conditions, the resource-building function of moral pride may be more readily realized, allowing this discrete positive emotion to contribute to broader positive affective experiences. In contrast, when burnout is high, depleted psychological resources may constrain leaders’ capacity to sustain and capitalize on positive experiences (Boley, 2025; Orazi et al., 2021). Accordingly, the positive relationship between moral pride and broader positive affect is expected to be weaker under high burnout than under low burnout.

The pattern may differ, however, for negative affect. Conservation of Resources Theory emphasizes not only resource accumulation but also the protection of remaining resources under conditions of scarcity (Hobfoll, 1989). When burnout is high, leaders are more vulnerable to negative affective experiences because their psychological and emotional resources are already depleted (Higson-Sweeney et al., 2024). Under such conditions, positive self-relevant experiences may assume greater protective significance as limited sources of psychological reinforcement (Bittner, 2023; Rodriguez & Kross, 2023; Scherer, 2022). Moral pride, by reinforcing a favorable evaluation of one’s own morally valued conduct, may therefore provide a comparatively more salient buffer against negative affect when burnout is high. When burnout is low and resources are relatively preserved, this protective function may still operate, but its incremental relevance may be less pronounced. Thus, whereas lower burnout may facilitate the resource-building function of moral pride for positive affect, higher burnout may increase the relative importance of its resource-protective function for negative affect. Accordingly, burnout may represent a resource-related condition under which the indirect relationships between ethical leadership behavior and broader affective experiences through moral pride manifest differently.

In summary, Figure 1 presents the overarching conceptual framework examined across the three studies, illustrating how ethical leadership behavior is associated with leaders’ emotional experiences through moral pride and how this process may vary as a function of stable individual differences and dynamic resource states.

To examine this conceptual framework, we conducted three separate but complementary studies. Study 1 used an experiment to examine the relationship between ethical/unethical leadership behavior and moral pride and the moderating role of CSE. Study 2 used a two-wave field survey to examine the mediating role of moral pride and the moderating role of CSE. Study 3 used an experience sampling design to explore whether the indirect relationships between ethical leadership behavior and leaders’ emotions via moral pride exhibit different conditional patterns across levels of burnout. Together, the three studies provide complementary experimental, field, and experience-sampling evidence for the proposed framework.

## 2. Study 1: The Effects of Ethical Leadership Behavior and Core Self-Evaluation on Moral Pride

### 2.1. Method

Using the professional online panel Credamo, we obtained a sample of 205 full-time employees located in China, who completed the study in Chinese. A priori statistical power analysis conducted with G*Power 3.1.9.7 demonstrated that, given an alpha level of 0.05 and anticipated effect size of 0.80, the obtained sample size achieved 95.47% statistical power to detect the hypothesized effects.

Participants were randomly assigned to one of three experimental conditions: ethical leadership behavior condition (*n* = 68), unethical leadership behavior condition (*n* = 65), and control condition (*n* = 72). Among the participants, 88 were male and 117 were female, with a mean age of 32.35 years (*SD* = 6.60).

Preliminary analyses confirmed that there were no significant differences across the three conditions in terms of age, *F*(2, 202) = 0.03, *p* = 0.969, η^2^ = 0.00; gender, χ^2^(2) = 5.54, *p* = 0.063, φ = 0.16; education level, χ^2^(6) = 5.52, *p* = 0.479, *V* = 0.12; or years of work experience, *F*(2, 202) = 0.03, *p* = 0.970, η^2^ = 0.00.

This experiment adopted a single-factor between-subjects design. The independent variable was leadership condition (ethical leadership behavior/unethical leadership behavior/control). The moderator was core self-evaluation, and the dependent variable was moral pride.

After providing informed consent, participants first completed a demographic questionnaire capturing key background variables (gender, age, educational attainment, and organizational tenure). Core self-evaluation was assessed prior to exposure to the experimental manipulation, given its conceptualization as a relatively stable personality trait. They were then presented with a written scenario adapted from prior experimental research on ethical and unethical leader decision making, as follows: “Imagine you are the head of a department in a company. This year, your department has only one promotion slot. Based on objective and fair criteria such as performance and potential, you should promote Employee A. However, you are not personally close to Employee A. In contrast, Employee B has weaker performance and potential, but you have a closer personal relationship with them and know them well. After a night of consideration, you decide that—”

Participants were randomly assigned to one of the three experimental conditions. In the ethical leadership behavior condition, the decision presented was: “You decide to recommend Employee A because it is a fair and just choice.” In the unethical leadership behavior condition, the decision was: “You decide to recommend Employee B—not because their performance and capability are the best—but because you have the closest relationship with them.” In the control condition, participants read: “The promotion decision this year is too difficult, so you decide to postpone it for a while.” This scenario was designed to manipulate the ethicality of a leadership decision: choosing Employee A represented a fair and ethically appropriate decision based on objective criteria, whereas choosing Employee B represented an unethical decision based on personal relationships rather than objective performance criteria.

After reading the scenario, participants first completed the manipulation check, followed by a measure of moral pride. Moral pride was assessed using a scale adapted from Boezeman and Ellemers (2007), which included items such as “I feel proud to be a moral person.” Core self-evaluation was measured using the instrument developed by Judge et al. (2003), with representative items including “I believe I will be successful in life” and “I am capable of handling most of my problems.”

Lastly, a manipulation check was conducted to verify the effectiveness of the ethical leadership manipulation. Drawing from Lin et al. (2016), two items were designed to assess participants’ perceived ethicality of the behavior: “Do you believe your decision was fair and just?” and “Do you believe your decision was ethical and moral?” Participants rated each item on a 5-point Likert scale (1 = strongly disagree, 5 = strongly agree), with higher scores indicating greater perceived ethicality.

### 2.2. Results

Significant group differences were found in participants’ ratings of the fairness and ethicality of the decision, confirming the effectiveness of the experimental manipulation. Specifically, participants in the ethical leadership behavior condition rated the decision as significantly more fair and ethical (*M* = 4.40 and 4.38, respectively) compared to those in the control condition (*M* = 3.14 and 3.33) and the unethical leadership behavior condition (*M* = 1.97 and 2.40), with all differences statistically significant (*F*(2, 202) = 104.90, *p* < 0.001, η^2^ = 0.51 for fairness; *F*(2, 202) = 53.38, *p* < 0.001, η^2^ = 0.35 for ethicality). Additionally, ratings in the unethical leadership behavior condition were significantly lower than those in the control condition (*p* < 0.001), indicating that participants clearly distinguished among the experimental conditions in terms of perceived ethical leadership behavior.

A regression analysis was conducted with moral pride as the dependent variable, ethical leadership behavior condition (ethical/unethical/control) as the independent variable, and core self-evaluation as a moderator. Dummy variables were created to contrast the conditions (D1: control vs. ethical; D2: control vs. unethical). The results showed that the coefficient for D1 was not significant (*B* = 0.09, *SE* = 0.09, *p* > 0.05), whereas the coefficient for D2 was significant and negative (*B* = −0.28, *SE* = 0.095, *p* = 0.004), indicating that participants in the unethical leadership behavior condition reported significantly lower levels of moral pride (*M* = 4.03) than those in the control condition (*M* = 4.26) and the ethical leadership behavior condition (*M* = 4.35). No significant difference in moral pride was found between the control and ethical leadership behavior groups. Taken together, these results indicate that the experimental effect was primarily driven by the lower level of moral pride in the unethical leadership behavior condition, whereas the ethical leadership behavior condition did not significantly differ from the control condition.

Regarding the moderation effect (Table 1), the interaction between D1 and core self-evaluation was not significant (*B* = 0.292, *SE* = 0.166, *p* > 0.05), whereas the interaction between D2 and core self-evaluation was significant (*B* = 0.385, *SE* = 0.179, *p* = 0.033). This suggests that the moderating effect of core self-evaluation was evident in the comparison between the unethical leadership behavior condition and the control condition. The overall regression model explained 18% of the variance in moral pride (*R*^2^ = 0.18), with a medium effect size (*f*^2^ = 0.21).

To further examine the direction of the moderating effect of core self-evaluation, simple slope analyses (Preacher, 2015) were conducted. Results indicated that when core self-evaluation was low (one standard deviation below the mean), moral pride in both the ethical leadership behavior group (*p* = 0.004) and the control group (*p* < 0.001) was significantly higher than that in the unethical leadership behavior group. When core self-evaluation was at the mean level, moral pride in the ethical leadership behavior group (*p* < 0.001) and the control group (*p* = 0.004) remained significantly higher than in the unethical leadership behavior group. When core self-evaluation was high (one standard deviation above the mean), moral pride in the ethical leadership behavior group was still significantly higher than in the unethical leadership behavior group (*p* = 0.019). See Figure 2 for details.

## 3. Study 2: The Effect of Ethical Leadership Behavior on Positive and Negative Emotions: A Moderated Mediation Model

### 3.1. Method

Building on Study 1, Study 2 examined the proposed process in a field setting by testing whether leaders’ ethical leadership behavior relates to their subsequent emotional experiences through moral pride, and whether the relationship between ethical leadership behavior and moral pride varies as a function of core self-evaluation.

This study adopted a two-wave survey design involving middle managers across diverse enterprises in China, spanning manufacturing, services, and technology sectors. The two waves were administered approximately 20 days apart. Participants were recruited through MBA alumni networks at a large university in China and received modest monetary incentives for completing each wave of the survey. In Wave 1, 161 managers were surveyed, and 147 provided valid responses (effective response rate = 91.30%). Of these managers, 132 provided valid responses in Wave 2, resulting in a final matched sample of 132 managers. All variables included in the analyses reported in the present study were assessed from the participating managers.

Ethical leadership behavior was measured in the Wave 1 leader survey using the eight-item scale adapted from Lin et al. (2016), with a Cronbach’s α of 0.702. Moral pride was assessed in the Wave 2 leader survey using the three-item scale adapted from Boezeman and Ellemers (2007), with a Cronbach’s α of 0.840. Positive and negative emotions were measured in the Wave 2 leader survey using the PANAS developed by Watson et al. (1988), with Cronbach’s α values of 0.894 and 0.939, respectively. Core self-evaluation was measured in the Wave 2 leader survey using the 12-item scale developed by Judge et al. (2003), with a Cronbach’s α of 0.895. All measures used a five-point Likert-type response format, with higher scores indicating higher levels of the respective constructs.

Among the 132 valid manager responses in the second wave, 80 were male and 52 female; ages ranged from 24 to 52 years, with a mean age of 32.58 (*SD* = 5.00). Regarding educational background, the majority held associate or bachelor’s degrees (53.79%) or master’s degrees (40.91%). More than 40% of respondents had held managerial positions for over three years. Detailed demographic characteristics are presented in Table 2.

Attrition analyses were conducted to assess potential biases between respondents who dropped out after the first wave and those who remained. No significant differences were found between these groups in terms of gender (χ^2^(1) = 0.21, *p* = 0.648, φ = 0.04), educational level (χ^2^(3) = 1.42, *p* = 0.702, φ = 0.10), or tenure in managerial positions (χ^2^(3) = 0.46, *p* = 0.928, φ = 0.06).

All statistical analyses were performed using SPSS 22.0 for descriptive statistics, correlations, regression, ANOVA, and reliability tests. Construct validity was assessed via confirmatory factor analysis in Mplus 7.2 including all focal variables in a single measurement model. Hayes’ (2017) PROCESS macro tested mediation and moderated mediation effects.

### 3.2. Results

Table 3 presents the means, standard deviations, and correlation coefficients of the main variables. Results show that ethical leadership behavior was significantly positively correlated with moral pride (*r* = 0.30, *p* < 0.001), and moral pride was significantly correlated with positive emotions (*r* = 0.47, *p* < 0.001) and negative emotions (*r* = −0.39, *p* < 0.001). These correlations provide preliminary evidence of associations among ethical leadership behavior, moral pride, and leaders’ broader affective experiences.

Since the data in this study were collected from the same source, common method bias (CMB) may be a concern. To address this, Harman’s single-factor test was conducted using confirmatory factor analysis (CFA).

For positive emotions, the single-factor model demonstrated poor fit indices: χ^2^ = 1245.94, df = 495, χ^2^/df = 2.52, CFI = 0.63, TLI = 0.60, RMSEA = 0.11, SRMR = 0.10. The four-factor model (ethical leadership behavior, moral pride, positive emotions, and core self-evaluation) showed better fit: χ^2^ = 838.83, df = 489, χ^2^/df = 1.72, CFI = 0.83, TLI = 0.81, RMSEA = 0.07, SRMR = 0.07. These results indicate that the four-factor model fits the data better than the single-factor model, suggesting that a single factor does not adequately account for the covariance among the measures and providing evidence for the distinctiveness of the four constructs.

For negative emotions, the single-factor model also demonstrated poor fit: χ^2^ = 1633.12, df = 495, χ^2^/df = 3.30, CFI = 0.54, TLI = 0.51, RMSEA = 0.13, SRMR = 0.14. The four-factor model (ethical leadership behavior, moral pride, negative emotions, and core self-evaluation) exhibited improved fit: χ^2^ = 928.49, df = 489, χ^2^/df = 1.90, CFI = 0.82, TLI = 0.81, RMSEA = 0.08, SRMR = 0.08. These results similarly indicate that the four-factor model fits the data better than the single-factor model, providing further evidence for the distinctiveness of the four constructs.

**Mediating role of moral pride.** The mediating effect of moral pride on the relationship between ethical leadership behavior and positive emotions was tested following the mediation analysis steps proposed by Wen and Ye (2014). First, multiple regression analysis showed that ethical leadership behavior significantly predicted positive emotions, supporting the total effect, *c* = 0.27, *SE* = 0.12, *p* = 0.024. Second, ethical leadership behavior significantly predicted moral pride, *a* = 0.42, *SE* = 0.12, *p* < 0.001; and when both ethical leadership behavior and moral pride were included as predictors of positive emotions, moral pride remained a significant predictor, *b* = 0.46, *SE* = 0.08, *p* < 0.001. Both the a and b paths were significant, providing initial evidence consistent with the proposed mediating role of moral pride. The indirect effect (*ab*) was 0.19, with a bootstrap *SE* of 0.07 and a 95% bootstrap confidence interval (CI) of [0.09, 0.35], excluding zero. Third, when both ethical leadership behavior and moral pride were included in the regression predicting positive emotions, the direct effect of ethical leadership behavior became non-significant, *c*′ = 0.08, *SE* = 0.11, *p* = 0.48. The mediation proportion was *ab*/*c* = 0.71, and the mediation ratio was *ab*/*c*′ = 2.43. Thus, the results support a significant positive indirect effect of ethical leadership behavior on positive emotions through moral pride. Detailed results are presented in Table 4 and Figure 3. These results indicate that ethical leadership behavior is positively associated with moral pride, which in turn is associated with more positive subsequent emotional experiences.

**The mediating role of moral pride in the relationship between ethical leadership behavior and negative emotions**. Following the steps for testing mediation effects, (1) a multiple regression analysis was conducted with negative emotions as the dependent variable and ethical leadership behavior as the independent variable. The result indicated that the coefficient of ethical leadership behavior was not significant, supporting a suppressor effect, with a total effect of *c* = −0.06, *SE* = 0.17, *p* = 0.720. (2) Ethical leadership behavior significantly predicted moral pride, *a* = 0.42, *SE* = 0.12, *p* < 0.001, and in the regression analysis with both ethical leadership behavior and moral pride predicting negative emotions, moral pride significantly predicted negative emotions, *b* = −0.55, *SE* = 0.12, *p* < 0.001. Both paths a and b were significant, providing initial evidence consistent with the proposed mediating role of moral pride. The indirect effect *ab* was −0.23, with a bootstrap-estimated *SE* = 0.10 and a 95% bootstrap confidence interval of [−0.48, −0.08], which did not include zero. (3) In the regression including both ethical leadership behavior and moral pride, the direct effect of ethical leadership behavior remained non-significant, *c*′ = 0.17, *SE* = 0.17, *p* = 0.312. Thus, the results support a significant negative indirect effect of ethical leadership behavior on negative emotions through moral pride.

From an identity-based perspective, these findings suggest that the association between ethical leadership behavior and lower negative emotions operates through the self-affirming experience of moral pride. Ethical leadership behavior is positively associated with moral pride, which in turn is negatively associated with subsequent negative emotional experiences. See Table 5 and Figure 4 for details.

**The moderating role of core self-evaluation.** According to Hayes’ (2017) procedure for testing moderated mediation, in the positive emotion model, (1) a multiple regression analysis was conducted with moral pride as the dependent variable and ethical leadership behavior, core self-evaluation, and their interaction term (Int: Ethical Leadership Behavior × Core Self-Evaluation) as predictors. The interaction term was significant, *B* = −0.452, *SE* = 0.190, *p* = 0.019. (2) The index of moderated mediation for the positive emotion model was −0.103, with a bootstrap *SE* of 0.052 and a 95% bootstrap confidence interval of [−0.233, −0.024], which did not include zero. The moderated mediation effect was significant. Specifically, the relationship between ethical leadership behavior and moral pride varied across levels of core self-evaluation, indicating that core self-evaluation moderated the first stage of the indirect effect.

In the negative emotion model, (3) the index of moderated mediation was 0.158, with a bootstrap *SE* of 0.102 and a 95% bootstrap confidence interval of [0.020, 0.437], also excluding zero. This indicates a significant moderated mediation effect in the negative emotion model as well, with core self-evaluation significantly moderating the first-stage path of the indirect effect, such that the influence of ethical leadership behavior on moral pride depends on the level of core self-evaluation. These results are consistent with the proposed role of CSE in shaping self-evaluative sensitivity, such that the extent to which ethical leadership behavior is associated with moral pride varies across levels of CSE. See Table 6 for details.

To further examine the direction of the moderating effect of core self-evaluation, a simple slopes analysis (Preacher, 2015) was conducted. The results showed that when core self-evaluation was low (one standard deviation below the mean), a one-unit increase in ethical leadership behavior predicted a 0.59-unit increase in moral pride (*SE* = 0.16, *t* = 3.71, *p* < 0.001). When core self-evaluation was at the mean level, a one-unit increase in ethical leadership behavior predicted a 0.33-unit increase in moral pride (*SE* = 0.09, *t* = 3.66, *p* < 0.001). However, when core self-evaluation was high (one standard deviation above the mean), the effect was no longer significant; a one-unit increase in ethical leadership behavior only led to a 0.06-unit increase in moral pride (*SE* = 0.19, *t* = 0.34, *p* = 0.736). These findings indicate that the positive association between ethical leadership behavior and moral pride becomes weaker as CSE increases, such that the association is stronger among leaders with lower CSE, supporting Hypothesis 3. See Figure 5 for details.

## 4. Study 3: The Dynamic Effects of Ethical Leadership Behavior on Positive and Negative Emotions—Evidence from an Experience Sampling Method (ESM) Approach

### 4.1. Method

Study 3 employed the Experience Sampling Method (Larson & Csikszentmihalyi, 2014) with 64 corporate managers recruited from part-time MBA programs (final *N* = 52 after excluding participants with insufficient response rates). All participants were required to have experience leading teams of at least two members. Participants completed surveys at three fixed daily intervals (10:30 AM, 3:00 PM, and 8:30 PM) over 10 workdays, yielding up to 30 observations per participant. To facilitate data collection, a dedicated WeChat group was created for the study, through which participants received reminders at the three scheduled survey times each day, and at each prompt were directed to a unique Wenjuanxing survey link corresponding to that time window. Building on Studies 1–2, Study 3 examined these relationships in a dynamic work context, with particular attention to the role of job burnout in the indirect relationship between ethical leadership behavior and leaders’ emotional experiences through moral pride. All participants held actual managerial responsibilities, with team sizes ranging from 2 to 100 employees (*M* = 14, *SD* = 18.84). See Table 7 for details.

The study assessed five focal constructs: ethical leadership behavior, moral pride, positive emotions, negative emotions, and job burnout. Ethical leadership behavior, moral pride, and positive and negative emotions were assessed using the same measures as in Study 2. Job burnout was assessed using a state-adapted Chinese version of the MBI-GS (Maslach et al., 2001) to capture fluctuations in leaders’ burnout states across measurement occasions. Data were analyzed using Mplus 8.3. Given the repeated-measures structure of the ESM data, with measurement occasions nested within 52 leaders across 10 workdays, a two-level modeling approach was used to account for the nested structure of the observations.

To examine the role of job burnout in the indirect pathway, participants were categorized into relatively higher- and lower-burnout groups using a median split. Within each group, the effects of ethical leadership behavior on moral pride and of moral pride on positive and negative emotions were estimated separately, and the corresponding indirect effects were calculated.

### 4.2. Results

To examine the role of job burnout in the indirect pathway from ethical leadership behavior to emotions via moral pride, a multi-group analysis based on relatively lower- and higher-burnout groups was conducted. In the low-burnout group, ethical leadership behavior significantly predicted moral pride (*B* = 0.81, *SE* = 0.06, *p* < 0.001), which in turn significantly predicted higher levels of positive emotions (*B* = 0.38, *SE* = 0.05, *p* < 0.001) and lower levels of negative emotions (*B* = −0.16, *SE* = 0.08, *p* = 0.041).

The analysis of indirect effects showed that in the low-burnout group (Table 8), ethical leadership had a significant positive indirect effect on positive emotions through moral pride (IND_low = 0.31, *SE* = 0.04, *p* < 0.001), as well as a significant negative indirect effect on negative emotions (IND_low = −0.13, *SE* = 0.06, *p* = 0.047).

By contrast, in the high-burnout group (Table 9), ethical leadership behavior also significantly predicted moral pride (*B* = 0.78, *SE* = 0.10, *p* < 0.001). However, the predictive effect of moral pride on positive emotions was only marginally significant (*B* = 0.15, *SE* = 0.08, *p* = 0.057), whereas moral pride was significantly negatively associated with negative emotions (*B* = −0.29, *SE* = 0.09, *p* = 0.002). The analysis of indirect effects indicated that in the high-burnout group, the indirect effect of ethical leadership on positive emotions via moral pride was marginally significant (IND_high = 0.12, *SE* = 0.06, *p* = 0.077), whereas the indirect effect on negative emotions was significant (IND_high = −0.23, *SE* = 0.08, *p* = 0.007).

Taken together, these results revealed a pattern consistent with the proposed resource-based account. Under low burnout, moral pride was associated with higher positive emotions and lower negative emotions, with the indirect effect on positive emotions being particularly evident. Under high burnout, the relationship between moral pride and positive emotions did not reach statistical significance, whereas the negative indirect effect on negative emotions remained significant. Thus, the pattern of conditional indirect effects suggests that lower burnout may facilitate the resource-building function of moral pride for positive affect, whereas under higher burnout, moral pride may operate primarily as a resource-protective mechanism associated with reduced negative affect.

## 5. Discussion

The current research was motivated by an important question in the study of leadership and ethics: How and under what conditions does engaging in ethical leadership shape leaders’ own emotional experiences? Although a substantial body of research has documented how ethical leaders influence followers’ attitudes, behaviors, and well-being (Aunin et al., 2024), emerging research has begun to recognize that ethical leadership may also have important emotional consequences for leaders themselves (Z. Xu & Ju, 2024). Building on this emerging actor-centric perspective, the present research advances understanding of the underlying process by integrating moral identity and self-conscious emotion theories to explain how ethical conduct relates to leaders’ emotional experiences through moral pride and how this process varies across individual and resource conditions. Across three complementary studies, our findings consistently point to moral pride as an important self-referential emotional mechanism linking ethical leadership behavior to broader affective experiences, although the strength and form of this relationship varied across studies and conditions. In particular, Study 3 revealed different affective patterns across burnout levels: under lower burnout, moral pride was associated with positive emotional benefits, whereas under higher burnout, its association with reduced negative affect was particularly evident.

### 5.1. Theoretical Implications

First, our work extends prior actor-centered research on the emotional consequences of ethical leadership by further specifying the affective process through which leaders’ own ethical conduct relates to their emotional experiences (Z. Xu & Ju, 2024). Prior research has extensively documented the influence of ethical leadership on subordinates (Nassif et al., 2021; Rice et al., 2025) and highlighted the role of leaders’ core self-evaluation in shaping their impact on followers (Sun et al., 2023). Leaders with higher core self-evaluation are more likely to act as honest and trustworthy role models, whose behaviors inspire subordinates to emulate ethical conduct (Eldor, 2021; Schuhmacher et al., 2022) and to influence followers through guidance, empowerment, and encouragement (Khudozhnikova et al., 2026). Although ethical leadership at all organizational levels is important, direct supervisors exert the strongest influence on subordinates’ moral behavior (Greenbaum et al., 2021). At the same time, emerging actor-centered research has begun to examine the emotional experiences of leaders themselves (Yip & Lee, 2022; Z. Xu & Ju, 2024). Building on this emerging perspective, the present research focuses more specifically on moral pride as a self-conscious emotional mechanism linking ethical leadership behavior to broader positive and negative affect. Drawing on moral identity and self-conscious emotion theories, we propose that ethical conduct can generate moral pride when it is appraised as congruent with morally relevant self-standards. Across our studies, moral pride emerged as an important mechanism linking ethical leadership behavior to greater positive affect and lower negative affect. We further extend this account by examining ethical versus unethical behavior experimentally and by considering the role of burnout as a resource-related condition in leaders’ daily emotional experiences. Thus, our findings extend prior actor-centered research by specifying the emotional mechanism involved and highlighting individual and resource-related conditions associated with leaders’ affective experiences following ethical conduct.

Our findings also complement prior research showing that ethical conduct can have demanding consequences for leaders themselves. Lin et al. (2016), for instance, demonstrated that acting ethically can deplete leaders’ self-regulatory resources and contribute to emotional exhaustion. Alongside this line of research, our findings indicate that ethical leadership behavior can also be associated with beneficial emotional experiences through moral pride. Moreover, these emotional benefits are not uniform across leaders or situations. CSE shapes the extent to which ethical leadership behavior is associated with moral pride, whereas different affective patterns associated with moral pride were observed across levels of burnout. Together, these findings provide a more differentiated account of leaders’ emotional experiences following ethical conduct by specifying both the self-conscious emotional mechanism involved and the conditions under which its affective implications vary.

Second, this research further highlights the role of self-conscious emotions in understanding the emotional consequences of leadership behavior. Existing studies have examined broad affective categories such as positive and negative emotions (Bakker et al., 2023; X. M. Xu & Yu, 2021), as well as discrete emotions such as anger and envy in leadership processes (Saha et al., 2023; Y. Yu & Shi, 2024). Building on this literature, our findings highlight moral pride as a particularly relevant self-conscious emotion in the context of ethical leadership. Moral pride is theoretically distinct from broader positive affect because it arises from a favorable self-evaluation of one’s morally relevant conduct; at the same time, once experienced, it may function as an affective resource that supports broader positive affect and buffers against negative affect. This distinction helps clarify how a specific self-conscious emotion can serve as an intermediate emotional mechanism linking ethical conduct to more general affective experiences. Future research could extend this work by examining other self-conscious emotions, such as moral elevation, shame, and guilt, in ethical leadership contexts (Hong et al., 2025; Jeong et al., 2025). Examining these distinct emotions may provide a more nuanced understanding of the affective processes associated with leadership behavior.

Third, we clarify the roles of individual and resource-related conditions in this identity-based emotional pathway by distinguishing between a stable trait and a dynamic state. We find a stronger relationship between ethical leadership behavior and moral pride among leaders with lower core self-evaluations (CSE), who generally hold less positive and less stable views of themselves. This pattern is consistent with our premise that favorable self-relevant information arising from ethical conduct may be more salient and psychologically consequential for lower-CSE leaders, thereby strengthening their experience of moral pride. Burnout, as a state of resource depletion, was associated with different affective patterns involving moral pride. Under lower burnout, the relationship between moral pride and positive affect was particularly evident, whereas under higher burnout, its buffering association with negative affect was more prominent. This pattern is consistent with the proposed resource-building and resource-protective functions of moral pride. Collectively, these findings highlight the relevance of both relatively stable individual differences and dynamic resource states to the identity-based emotional pathway.

### 5.2. Practical Implications

From a practical perspective, this study offers several important managerial implications. First, our findings provide additional support for organizational efforts to encourage and sustain ethical leadership behavior. Prior research has demonstrated that ethical leadership positively influences subordinates (Amory et al., 2024; Luu, 2023; Nassif et al., 2021) and can create an altruistic moral climate that fosters employees’ moral identification and participation in helping behaviors, thereby benefiting the organization as a whole (Farooq & Khan, 2026). With the development of positive psychology and the rise in positive organizational behavior, increasing attention is being paid to employees’ emotional states and well-being. Given that leaders’ emotions are likely to be contagious to subordinates, affecting their mental and physical health as well as team performance (Xerri et al., 2023), the present findings suggest that ethical leadership behavior can also be associated with beneficial emotional experiences for leaders themselves through moral pride. Organizations may therefore consider incorporating the intrapersonal emotional implications of ethical conduct into leadership development and ethics training, helping leaders recognize that ethical leadership may contribute not only to positive outcomes for followers and organizations but also to their own emotional experiences.

Second, our findings have implications for supporting leaders with different levels of core self-evaluation and psychological resources. Individuals with low core self-evaluation tend to experience fewer positive emotions and more intense negative emotions (Peláez-Fernández et al., 2022). Such emotional states can be detrimental to their mental and physical health and are associated with lower work efficiency (Krauss & Orth, 2022), poorer job performance (Peláez-Fernández et al., 2022), and reduced leadership effectiveness (Mitchell et al., 2024). Our findings suggest that the relationship between ethical leadership behavior and moral pride is particularly pronounced among leaders with lower CSE. Thus, leadership development practices may help lower-CSE leaders recognize and reflect on the positive self-relevant meaning of their ethical conduct, potentially strengthening the moral pride associated with such behavior. Additionally, our findings highlight the importance of psychological resource management for leaders (Mo et al., 2025). Organizations may support leaders’ resource levels through stress-reduction practices and emotion-regulation training. Importantly, the emotional implications of moral pride appear to differ across levels of burnout: under lower burnout, its association with broader positive affect was particularly evident, whereas under higher burnout, its buffering association with negative affect was more pronounced. Accordingly, leadership support practices should recognize that psychological resource states may be relevant to how leaders derive emotional benefits from their ethical conduct.

### 5.3. Limitations and Future Research

This study has several noteworthy limitations that also suggest directions for future research. First, although our theoretical framework draws on moral identity theory and self-conscious emotion theory to explain why leaders’ ethical conduct may elicit moral pride, we did not directly measure leaders’ moral identity or their perceived self-congruence with moral standards. Accordingly, the proposed self-evaluative process should be interpreted as a theoretically grounded explanation rather than as a directly tested psychological mechanism. Future research could incorporate direct measures of moral identity centrality and perceived moral self-congruence to examine more explicitly when ethical conduct is experienced as self-affirming and subsequently gives rise to moral pride. Such research could also examine whether the strength of this process varies depending on the salience of leaders’ moral identity or across situations in which ethical conduct is more or less consistent with personally meaningful moral standards.

Second, the present research focuses primarily on individual-level and resource-related conditions, while giving less attention to broader contextual factors that may shape leaders’ emotional responses to their own ethical conduct. Prior research suggests that contextual conditions such as work pressure can influence the consequences of ethical leadership (Schwepker & Dimitriou, 2021). Moreover, in organizational environments characterized by political dynamics or strong impression-management concerns, the meaning that leaders attach to ethical behavior may differ (Sarwar et al., 2023). Such contextual factors may influence whether ethical conduct is experienced as personally meaningful and self-affirming and, consequently, the extent to which it gives rise to moral pride and subsequent affective experiences. Future research should therefore examine organizational and situational contingencies alongside individual characteristics to further specify the conditions under which the emotional consequences examined here are more or less likely to emerge.

Third, common method variance remains a potential concern in Studies 2 and 3, particularly because the focal mediator and emotional outcomes relied on self-reported measures. We attempted to mitigate this concern through temporal separation and, in Study 3, repeated experience-sampling assessments, but shared-method and response biases cannot be ruled out entirely, especially for morally laden constructs (H. Yu et al., 2025). In addition, the measurement properties of Study 2 warrant some caution. The measurement model showed acceptable absolute fit on RMSEA and SRMR but fell short on CFI and TLI. While the four-factor model showed better fit than the single-factor model and provided evidence for the distinctiveness of the focal constructs, the measurement model should be treated as adequate rather than optimal. Future research should validate this structure with refined measures. In addition, Study 2 revealed a significant negative indirect effect of ethical leadership behavior on negative emotions through moral pride, despite a nonsignificant total effect and a nonsignificant residual direct effect. This pattern suggests that moral pride may represent one pathway through which ethical leadership behavior relates to negative affect, while other unmeasured psychological processes may also be involved. Future research could incorporate multi-source data or physiological indicators to reduce reliance on self-report, while also examining additional mechanisms that may operate alongside moral pride in shaping leaders’ negative emotional experiences.

## 6. Conclusions

In summary, this research extends prior actor-centered research on ethical leadership by examining how leaders’ own ethical conduct is associated with their emotional experiences through moral pride. Across three studies, the findings indicate that moral pride represents an important self-conscious emotional mechanism linking ethical leadership behavior to leaders’ broader affective experiences. Individual and resource-related conditions also emerge as relevant to these relationships: core self-evaluation shapes the relationship between ethical leadership behavior and moral pride, whereas different affective patterns associated with moral pride were observed across levels of burnout. Taken together, the findings further specify the emotional mechanism underlying the intrapersonal emotional consequences of ethical leadership behavior while highlighting the relevance of individual and resource-related conditions.

## Figures and Tables

**Figure 1:**
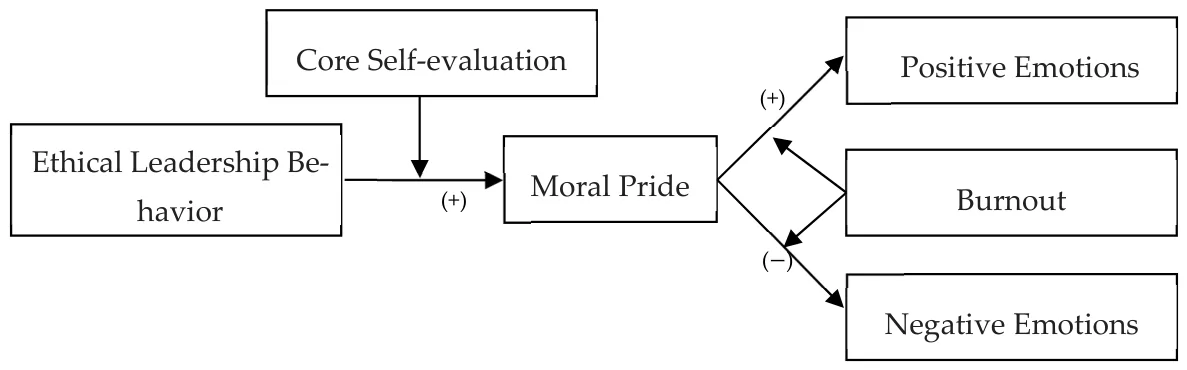
Theoretical framework.

**Figure 2:**
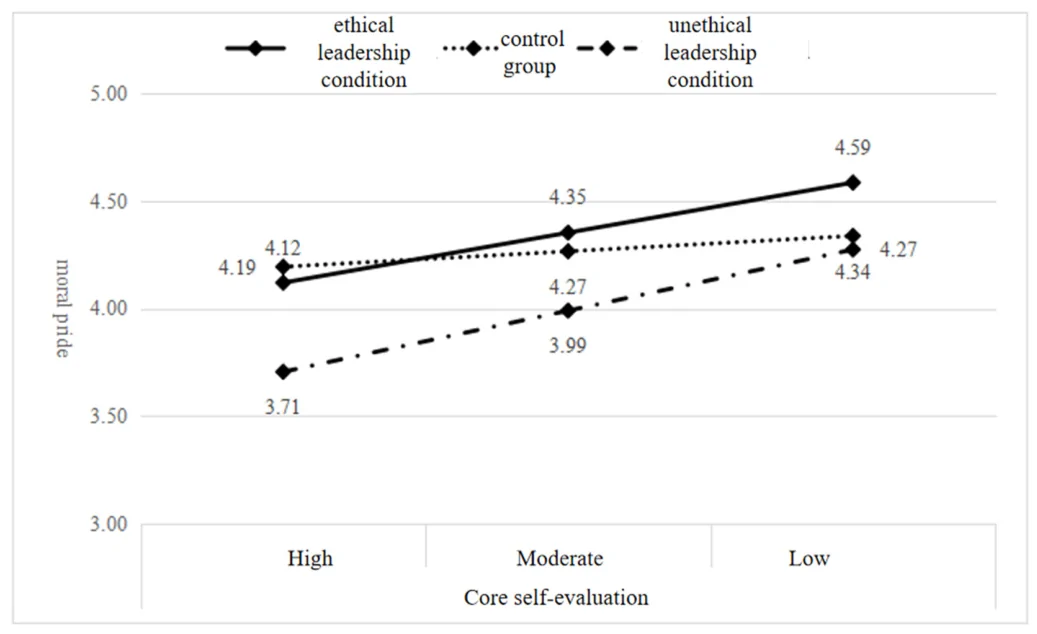
Moderating Effect of Core Self-Evaluation on the Relationship Between Ethical Leadership Behavior and Moral Pride.

**Figure 3:**
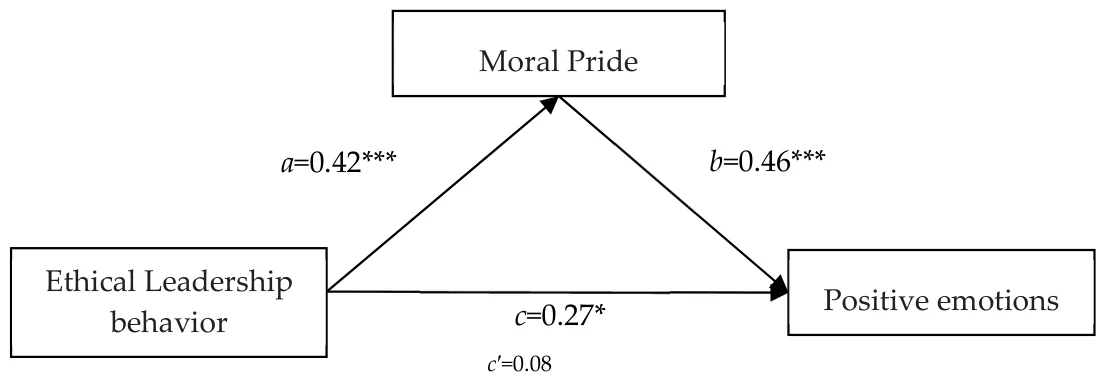
Mediation Model of Moral Pride in the Relationship Between Ethical Leadership Behavior and Positive Emotions. Note: * *p* < 0.05, *** *p* < 0.001.

**Figure 4:**
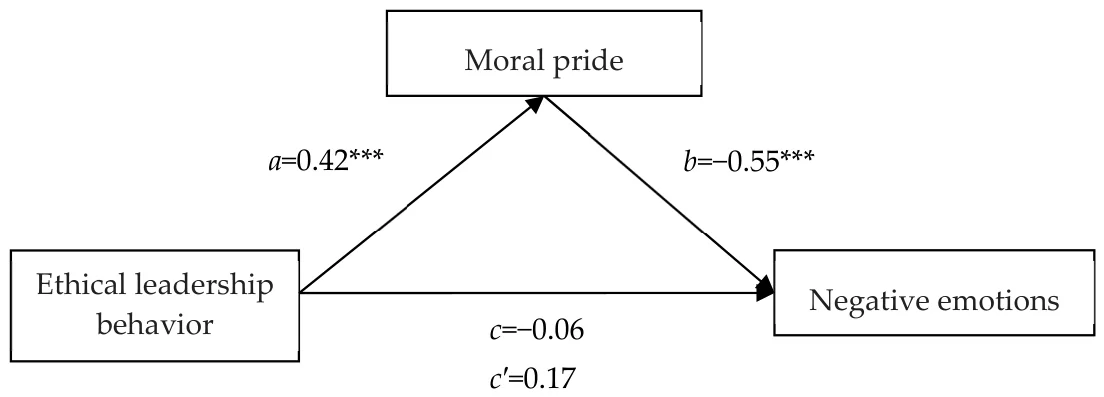
The Mediating Role of Moral Pride Between Ethical Leadership Behavior and Negative Emotions. Note: *** *p* < 0.001.

**Figure 5:**
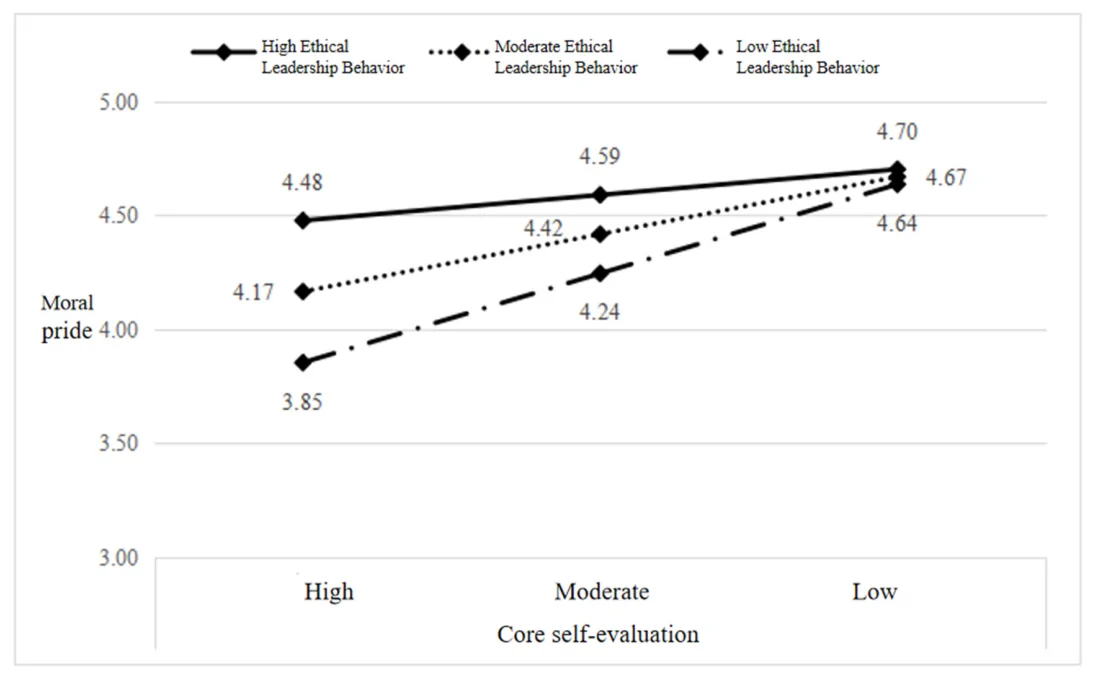
The Moderating Effect of Core Self-Evaluation on the Relationship Between Ethical Leadership Behavior and Moral Pride.

**Table 1:** Model: Moderating Effect of Core Self-Evaluation on the Relationship Between Ethical Leadership Behavior and Moral Pride.

	*B*	*SE*	*t*	*p*	95% CI
Constant	4.27	0.07	65.51	<0.001	[4.14, 4.39]
Core self-evaluation	0.13	0.12	1.08	0.283	[−0.11, 0.37]
D1	0.09	0.09	0.93	0.353	[−0.10, 0.27]
D2	−0.28	0.09	−2.91	0.004	[−0.46, −0.09]
D1 Core self-evaluation	0.29	0.17	1.76	0.080	[−0.04, 0.62]
D2 Core self-evaluation	0.39	0.18	2.15	0.033	[0.03, 0.74]

Note: Ethical leadership behavior was dummy-coded. D1 represents the comparison between the control group and the ethical leadership behavior group, and D2 represents the comparison between the control group and the unethical leadership behavior group.

**Table 2:** Sample distribution of leaders.

		Number of Participants	Percentage (%)
Gender	Male	80	60.61
	Female	52	39.39
Education level	High School or Below	5	3.79
	College/Bachelor’s Degree	71	53.79
	Master’s Degree	54	40.91
	Doctoral Degree	2	1.52
Years in managerial position	Less than 6 months	25	18.94
	6 months to 1 year	14	10.61
	1–3 years	32	24.24
	More than 3 years	61	46.21
Total		132	100.00

**Table 3:** Descriptive Statistics and Correlations of Key Variables.

	Mean	Standard Deviation	Correlation Coefficient			
			Ethical Leadership Behavior	Moral Pride	Positive Emotions	Negative Emotions
Ethical leadership behavior	4.00	0.40				
Moral pride	4.40	0.60	0.30 ***			
Positive emotions	3.80	0.60	0.19 *	0.47 ***		
Negative emotions	2.10	0.80	−0.03	−0.39 ***	−0.30 ***	
Core self-evaluation	3.90	0.50	0.22 *	0.46 ***	0.66 ***	−0.47 ***

Note: * *p* < 0.05, *** *p* < 0.001.

**Table 4:** The Mediating Role of Moral Pride Between Ethical Leadership Behavior and Positive Emotions.

	Outcome Variable(s)					
	Positive Emotions		Moral Pride		Positive Emotions	
	*B*	*SE*	*B*	*SE*	*B*	*SE*
Constant	2.48 ***	0.66	1.72 **	0.64	1.69 **	0.61
Gender	0.08	0.10	0.13	0.10	0.02	0.09
Age	−0.00	0.01	0.02	0.01	−0.01	0.01
Education level	0.10	0.09	0.08	0.08	0.06	0.08
Years in Managerial Role	−0.00	0.05	0.03	0.05	−0.02	0.04
Ethical leadership behavior	0.27 *	0.12	0.42 ***	0.12	0.08	0.11
Moral pride	—	—	—	—	0.46 ***	0.08
Model	*R*^2^ = 0.05		*R*^2^ = 0.14		*R*^2^ = 0.24	
	*F*(5, 126) = 1.45		*F*(5, 126) = 3.94 **		*F*(6, 125) = 6.55 ***	
	*f*^2^ = 0.06		*f*^2^ = 0.16		*f*^2^ = 0.31	

Note: * *p* < 0.05, ** *p* < 0.01, *** *p* < 0.001.

**Table 5:** The Mediating Role of Moral Pride Between Ethical Leadership Behavior and Negative Emotions.

	Outcome Variable(s)					
	Negative Emotions		Moral Pride		Negative Emotions	
	*B*	*SE*	*B*	*SE*	*B*	*SE*
Constant	3.74 ***	0.96	1.72 **	0.64	4.69 ***	0.92
Gender	0.02	0.15	0.13	0.10	0.09	0.14
Age	−0.03	0.02	0.02	0.01	−0.02	0.02
Education level	−0.10	0.12	0.08	0.08	−0.06	0.12
Years in Managerial Role	−0.06	0.07	0.03	0.05	−0.04	0.07
Ethical leadership behavior	−0.06	0.17	0.42 ***	0.12	0.17	0.17
Moral pride	—	—	—	—	−0.55 ***	0.12
Model	*R*^2^ = 0.06		*R*^2^ = 0.14		*R*^2^ = 0.19	
	*F*(5, 126) = 1.53		*F*(5, 126) = 3.94 **		*F*(6, 125) = 4.79 ***	
	*f*^2^ = 0.06		*f*^2^ = 0.16		*f*^2^ = 0.23	

Note: ** *p* < 0.01, *** *p* < 0.001.

**Table 6:** Moderated Mediation Model of the Effect of Core Self-Evaluation on the Relationship Between Ethical Leadership Behavior and Moral Pride.

	Outcome Variable(s)					
	Model Pride		Positive Emotions		Negative Emotions	
	*B*	*SE*	*B*	*SE*	*B*	*SE*
Constant	3.94 ***	0.39	3.38 ***	0.43	4.15 ***	0.76
Gender	0.13	0.09	0.05	0.08	0.07	0.13
Age	0.01	0.01	−0.01	0.01	−0.02	0.01
Education level	0.00	0.08	−0.00	0.06	−0.00	0.11
Years in managerial position	−0.01	0.04	−0.06	0.04	0.00	0.06
Ethical leadership behavior	0.33 **	0.11	0.00	0.09	0.24	0.16
Core self-evaluation	0.43 ***	0.09	0.68 ***	0.08	−0.59 ***	0.14
Int	−0.45 *	0.19	0.10	0.16	−0.04	0.28
Model pride			0.23 **	0.07	−0.35 **	0.13
Model	*R*^2^ = 0.30		*R*^2^ = 0.52		*R*^2^ = 0.29	
	*F*(7, 124) = 7.59 ***		*F*(8, 123) = 16.53 ***		*F*(8, 123) = 6.23 **	
	*f*^2^ = 0.43		*f*^2^ = 1.08		*f*^2^ = 0.41	

Note:* *p* < 0.05, ** *p* < 0.01, *** *p* < 0.001. Int refers to the interaction term between ethical leadership behavior and core self-evaluation.

**Table 7:** Sample distribution of leaders.

		Number of Participants	Percentage (%)
Gender	Male	10	19.2
	Female	42	80.8
Number of team members led	10 or fewer	34	65.4
	11–30	11	21.2
	31 or more	7	13.4
Tenure in current position	1 year or less	8	15.4
	1–5 years	20	38.5
	5–10 years	15	28.8
	More than 10 years	9	17.3
Total		52	100.00

**Table 8:** The mediating pathway of ethical leadership behavior on emotions via moral pride.

	Outcome Variable(s)					
	Moral Pride		Positive Emotions		Negative Emotions	
	*B*	*SE*	*B*	*SE*	*B*	*SE*
Constant	0.77 **	0.28	1.89 ***	0.20	2.40 ***	0.31
Ethical leadership behavior	0.81 ***	0.06	—	—	—	—
Moral pride	—	—	0.38 ***	0.05	−0.16 *	0.08

Note: * *p* < 0.05, ** *p* < 0.01, *** *p* < 0.001.

**Table 9:** The mediating pathway of ethical leadership behavior on emotions via moral pride.

	Outcome Variable(s)					
	Moral Pride		Positive Emotions		Negative Emotions	
	*B*	*SE*	*B*	*SE*	*B*	*SE*
Constant	0.85 *	0.39	2.86 ***	0.34	2.94 ***	0.42
Ethical leadership behavior	0.78 ***	0.10	—	—	—	—
Moral pride	—	—	0.15	0.08	−0.29 **	0.09

Note: * *p* < 0.05, ** *p* < 0.01, *** *p* < 0.001.

## Data Availability

The data presented in this study are available from the corresponding author upon request. The data are not publicly available due to privacy and ethical considerations related to the research participants.
